# Potential Beneficial Effects of Si-Wu-Tang on White Blood Cell Numbers and the Gastrointestinal Tract of γ-Ray Irradiated Mice

**Published:** 2014-09

**Authors:** Jin Ni, Ana L. Romero-Weaver, Ann R. Kennedy

**Affiliations:** 1 Department of Radiation Oncology, University of Pennsylvania Perelman School of Medicine, Philadelphia, Pennsylvania 19104, USA;; 2 Current Address: Department of Radiation Medicine, Faculty of Naval Medicine, Second Military Medical University, Shanghai, China

**Keywords:** Si-Wu-Tang, gamma radiation, white blood cells, lymphocytes, granulocytes, monocytes, gastrointestinal tract, fibrinogen

## Abstract

Si-Wu-Tang (SWT) is a decoction consisting of a mixture of ingredients of Rehmanniae Radix, Angelica Radix, Chuanxiong Rhizoma and Paeoniae Radix. As a traditional Chinese herbal decoction, SWT has been widely used for the treatment of diseases characterized as blood and/or energy deficit. The present study was performed to evaluate the effects of SWT on the different populations of circulating white blood cells (WBCs) and gastrointestinal changes in γ-ray irradiated mice. Female mice were treated daily with orally administered SWT seven days before irradiation, until one day before irradiation or until one day before sample collection. WBC counts were determined from peripheral blood samples taken from the mice at different times post-irradiation. Hematoxylin and eosin (H&E) staining, as well as immunohistochemical analysis of fibrinogen, were utilized to evaluate the effects of SWT in the intestines of mice after radiation exposure. The results of the present studies demonstrate that SWT has protective effects against radiation damage to circulating WBCs, specifically to lymphocytes, and to the gastrointestinal tract of the irradiated animals.

## INTRODUCTION

The effects of space radiation on the health of astronauts during space travel are of particular concern for manned missions in space. Radiation exposure in space, either from protons in solar particle events (SPEs) or SPE protons accompanied by high-mass, high atomic-number (Z) and high-energy particles known as HZE particles, may result in radiation sickness, with symptoms such as fatigue, hematopoietic malfunction due to immune system depression, chronic infection and diarrhea (1). Equally concerning is the radiation sickness resulting from radiation therapy or accidental exposure to radiation on earth. A variety of studies have been conducted over the past decades to find countermeasures that can mitigate the health risks to astronauts imposed by space radiation exposure, such as reduced leukocyte counts followed by immune system deficiency, that may jeopardize a deep-space mission (2, 3) as well as to mitigate radiation damage resulting from radiotherapy or accidental exposure (4). By comparison with pharmacological management, which typically involves injection of alcohols, sulfhydryls and/or cytokines shortly before irradiation to chemically mitigate the radiation damage, diet or nutritional countermeasures can be more advantageous since they can be administered orally before, during or after radiation exposure (5).

Herbal medicines have been commonly used in China to combat acute radiation injuries from radiotherapy or nuclear accidents (6). Si-Wu-Tang, (SWT), is a pharmaceutical grade traditional Chinese herbal formulation composed of ingredients of four herbal plants of Rehmanniae Radix, Angelica Radix, Chuanxiong Rhizoma and Paeoniae Radix (7). The use of SWT has been documented for thousands of years in traditional Chinese medicine for the treatment of diseases involving a variety of systems and tissues, including gynecological conditions, cutaneous inflammation, and hematopoiesis deficiency (8-10). The numbers of peripheral WBCs were previously shown to be increased by SWT treatment in animals irradiated with a low dose of ^60^Co γ –rays (7, 11, 12), but these studies did not address the specific populations of WBCs affected by SWT. Thus, the purpose of the present study was to evaluate the effect of SWT in each specific population of WBCs in mice irradiated at a dose of 2 Gy γ-rays.

The effects of SWT on the adverse effects of radiation in the gastrointestinal tract have not previously been reported. Thus, in addition to the effects of SWT on peripheral WBC counts, in the present study, the effectiveness of orally administered SWT at preventing or mitigating the adverse effects of radiation in the intestines of mice was also evaluated.

## MATERIALS AND METHODS

### Experimental Animals

Female ICR mice aged 6 weeks were purchased from Taconic Farms Inc. (Germantown, NY, USA). Animals were acclimated for one week in the University of Pennsylvania animal facility. Five animals were housed per cage with *ad libitum* access to water and food pellets. The animal care and experimental procedures were approved by the Institutional Animal Care and Use Committee of the University of Pennsylvania.

### Irradiation and SWT Administration

SWT (XSD Pharmacy Company, lot Number 120306, Chengdu, China), was administered using two different regimens. In one regimen mice were treated with SWT for 7 days prior to irradiation until one day before irradiation. A non-irradiated group and an irradiated group were treated with PBS at the same times that SWT treatments were performed to serve as controls. In the second group, mice were treated with SWT starting seven days prior to irradiation and continued after irradiation until one day before blood sample collection. Also, a non-irradiated group and an irradiated group were treated with PBS at the same time that SWT treatments were performed to serve as controls. For both regimes, blood samples were collected on days 4, 10, 13, 16, 18 and 20 post-irradiation. SWT and PBS were administered by daily oral gavages for the time periods indicated, in a total volume of 200 μl.

For irradiation, mice were exposed to a single dose, 2 Gy, of total body irradiation administered at a dose rate of 0.41 Gy/min using a ^137^Cs source (Gammacell 40 irradiator, Nordion, Ottawa, ON, Canada).

### Blood Sample Collection

Blood samples were collected at 4, 10, 13 16 and 18 days post-irradiation. At each time point, 5 animals per treatment group and per time point were euthanized by CO_2_ inhalation, and blood samples were collected by cardiac puncture, placed into lavender top blood BD microtainer collection tubes containing EDTA (BD, Franklin Lakes, NJ, USA) and sent to Antech Diagnostics facility (Lake Success, NY, USA) for automated complete blood cell count analysis. To control for possible fluctuations in blood cell counts associated with physical restraint, hormonal and/or circadian variations, blood samples from non-irradiated control mice and irradiated mice treated with PBS were collected at the same time points.

### Histology and Intestinal Mucosa Hematoxylin-Eosin Staining

Mice treated with PBS or SWT daily for seven days before irradiation until one day before irradiation were euthanized by CO_2_ inhalation on day-20 post-irradiation. Then, 0.5 cm size intestinal tissue samples were harvested from the ileum based on anatomical landmarks. The tissue samples were fixed in 10% neutral buffered formalin for 12 hours and then cut into small pieces and stored in 70% ethanol for paraffin embedding, which was followed by standard paraffin embedding procedures. From paraffin blocks, 5 μm thick sections were prepared for hematoxylin-eosin (H&E) staining. H&E stained sections were graded using light microscopy according to the appearance of cells showing condensed or fragmented nuclei and/or empty cytoplasm. The percentages of cells having different appearances/abnormalities, as described above, per 100 tubules, and the intestinal mucosal injury, were determined from three H&E stained sections from each animal. The sections were evaluated under a light microscope according to the criteria described by Chiu *et al*. (13) as follows: (-): normal, no appearance of mucinous cells, necrotic cells and cells with condensed nucleus; (+): mild, small number of mucinous cells, necrotic cells and cells with condensed nucleus; (+ +): moderate, medium number of mucinous cells, necrotic cells and cells with condensed nucleus; (+ + +): severe, large number of mucinous cells, necrotic cells and cells with condensed nucleus. All morphometric analyses were scored blindly with respect to experimental treatments by a single observer with expertise in mouse pathology. The mounted intestinal segments were photographed at 200× power magnification. The photomicrographs taken were optimized by Adobe Photoshop CS (San Jose, CA, USA) and evaluated for the intestinal mucous epithelial changes on ten randomly selected fields for each experimental group.

### Immunohistochemical Staining of Intestinal Fibrinogen

Fibrinogen immunohistochemical staining was performed on five intestinal sections per mouse. Paraffin sections were prepared in the same manner as described for the H&E processing. Then the sections were de-waxed and rehydrated; antigen retrieval and anti-fibrinogen antibody staining were carried out according to the manufacturer’s instructions (rabbit-antiserum, DAKO, Carpinteria. CA). The amount of fibrinogen immunofluorescence staining in each section was quantified using a modified version of a semi-automated method. Briefly, tissue sections were digitized using the Carl Zeiss Pro scanner (Thornwood, NY). Regions of low, medium and high levels of immunolabelling were selected by density thresholding of grey scale converted images using SigmaScan Pro5 image analysis software. Thresholds were selected by comparison of the staining intensity on intestinal sections taken from control animals using a light microscope equipped with a digital camera, and a computer-aided image capture system. The Pathological score of the fibrinogen immunostaining was determined using the NIH scoring system (14).

### Statistical Analyses

All statistical significance determinations reported in this manuscript were performed by using a Student’s *t*-test with Sigma Plot software.

## RESULTS

### Effects of SWT on Peripheral WBC Counts

**Figure 1:**
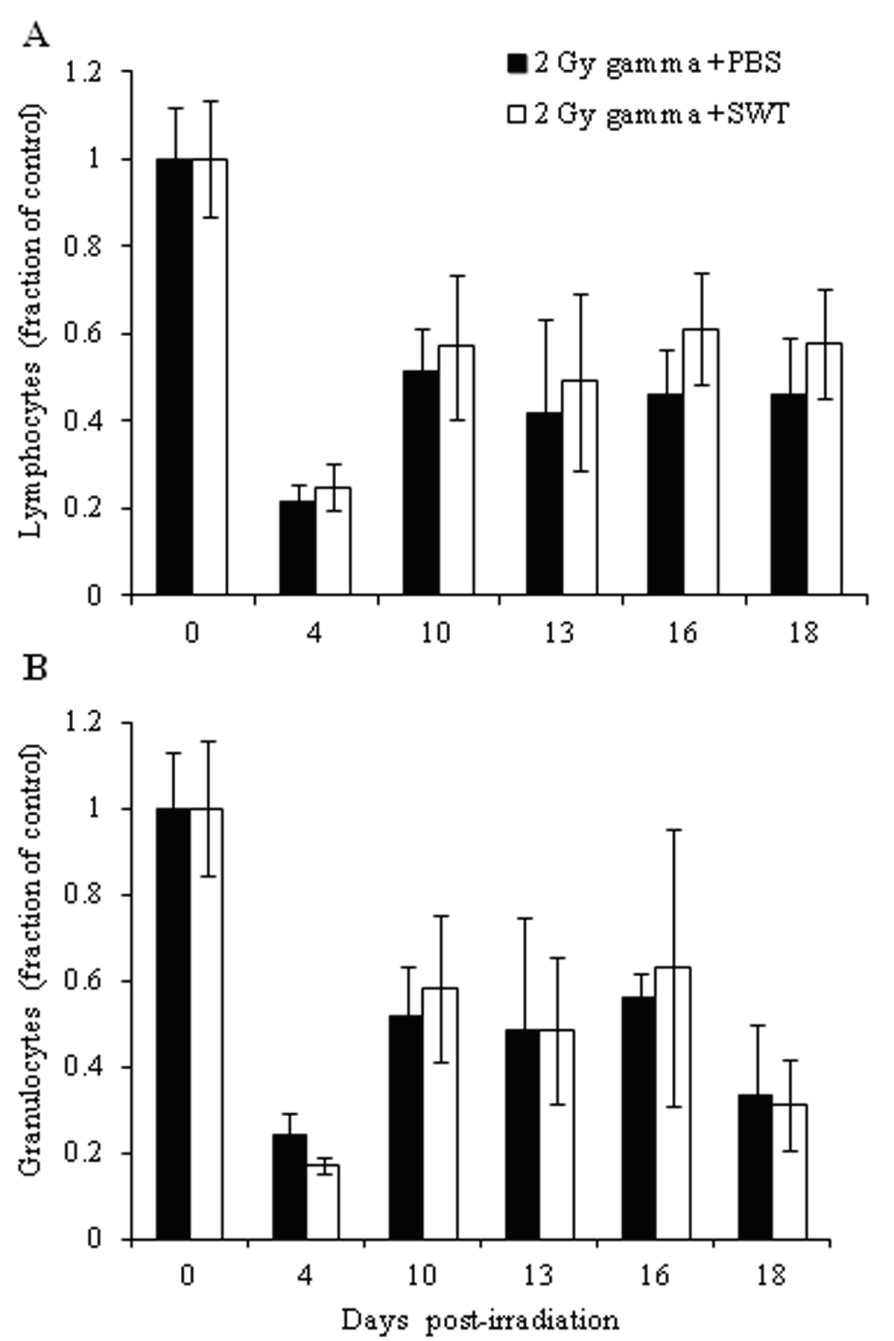
Direct comparison between Lymphocyte counts (A) and granulocyte counts (B) of 2 Gy gamma irradiated mice treated with PBS before and after irradiation (black bars) and 2 Gy gamma irradiated mice treated with SWT before and after irradiation (clear bars). The error bars represent the standard deviation.

Two regimens of SWT administration were evaluated. In the first regimen, total WBCs, lymphocytes, granulocytes and monocytes were counted at different time points in irradiated mice treated daily with SWT or PBS for 7 days before irradiation until one day before irradiation. Non-irradiated animals treated with PBS for the same time period were included in the experiment as a control group. Blood cell counts were determined at 4, 10, 13, 16 and 18 days post-irradiation. Significant decreases in WBC and lymphocyte counts were observed in irradiated mice treated with PBS at all of the time points analyzed (*p<0.05), as compared to the WBC and lymphocyte counts in the non-irradiated mice treated with PBS. Significant decreases were also observed in granulocyte counts on days 4, 10 and 18 and in monocyte counts on days 10, 13, 16 and 18 post-irradiation in the irradiated PBS-treated group, compared with the non-irradiated PBS-treated control group (Table 1). Irradiated animals treated with SWT exhibited statistically significant increases in WBC counts on days 4 and 18 post-irradiation, in lymphocyte counts on days 4, 10 and 18 post-irradiation, in granulocyte counts on days 4, 13, 16 and 18 post-irradiation and in monocyte counts on day 18 post-irradiation, compared with the corresponding blood cell counts of irradiated mice treated with PBS before irradiation; these counts were not significantly different from the corresponding counts in non-irradiated control mice treated with PBS (Table 1). A direct comparison of the most abundant types of white blood cells, lymphocytes and granulocytes, was made between irradiated mice treated with PBS and irradiated mice treated with SWT. The results shown in Figure 1 show that, although in general the differences between the numbers of peripheral lymphocytes and granulocytes in irradiated mice treated with SWT were higher than those in the irradiated mice treated with PBS, they were not statistically significant.

**Table 1:** Leukocyte counts determined in non-irradiated mice treated with PBS (PBS) and irradiated mice treated with PBS (IRR+PBS) or SWT (IRR+SWT) before irradiation

Cells (× 10^3^/μl)	Treatment	4 days	10 days	13 days	16 days	18 days

WBC	PBS	7.03 ± 2.19	13.73 ± 2.08	8.74 ± 2.52	7.40 ± 1.81	7.10 ± 2.15
	IRR+PBS	3.00 ± 0.64*	4.50 ± 1.10*	4.00 ± 0.66*	2.32 ± 0.54*	3.45 ± 1.24*
	IRR+SWT	4.00 ± 0.63	7.02 ± 1.88*	5.20 ± 1.28*	4.22 ± 1.35*	5.60 ± 0.67
Lymphocytes	PBS	5.40 ± 1.83	10.81 ± 2.60	6.82 ± 1.77	5.74 ± 1.2	5.60 ± 1.80
	IRR+PBS	2.00 ± 0.54*	3.45 ± 1.36*	3.10 ± 0.29*	1.92 ± 0.40*	2.62 ± 0.75*
	IRR+SWT	2.90 ± 0.54	5.55 ± 1.74	4.23 ± 1.22*	3.50 ± 1.10*	4.50 ± 0.50
Granulocytes	PBS	1.42 ± 0.41	2.70 ± 0.57	1.74 ± 0.77	1.49 ± 1.05	1.42 ± 0.43
	IRR+PBS	0.90 ± 0.13*	0.90 ± 0.28*	0.87 ± 0.36	0.39 ± 0.17	0.68 ± 0.50*
	IRR+SWT	0.93 ± 0.18	1.34 ± 0.32*	0.91 ± 0.35	0.70 ± 0.33	1.00 ± 0.35
Monocytes	PBS	0.21 ± 0.07	0.27 ± 0.04	0.17 ± 0.05	0.15 ± 0.07	0.12 ± 0.04
	IRR+PBS	0.16 ± 0.05	0.12 ± 0.03*	0.06 ± 0.06*	0.02 ± 0.01*	0.06 ± 0.06*
	IRR+SWT	0.15 ± 0.09	0.13 ± 0.04*	0.05 ± 0.01*	0.04 ± 0.01*	0.14 ± 0.12

* *p* values < 0.05 for comparison with non-irradiated mice treated with PBS.

**Figure 2:**
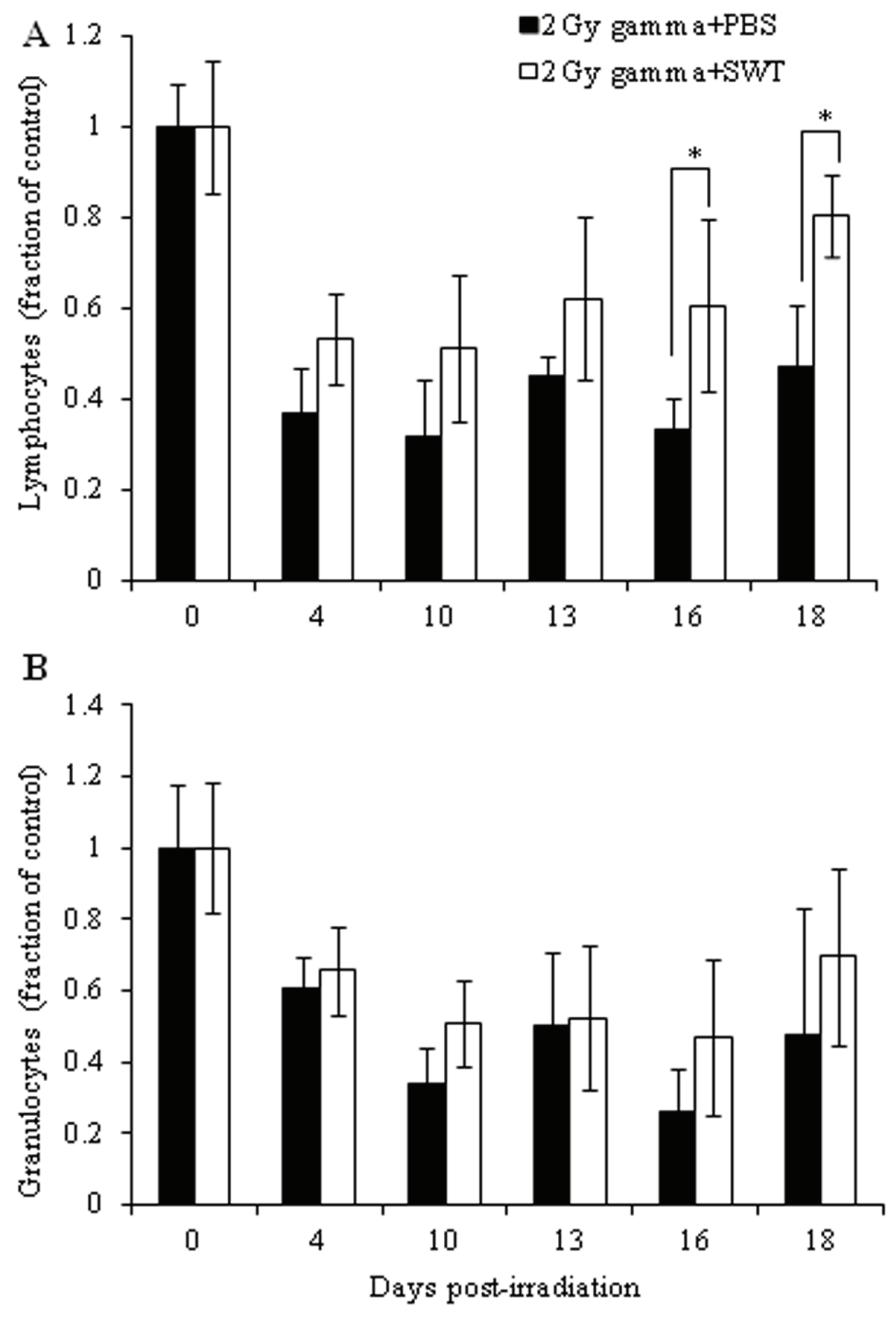
Direct comparison between Lymphocyte counts (A) and granulocyte counts (B) of 2 Gy gamma irradiated mice treated with PBS before irradiation (black bars) and 2 Gy gamma irradiated mice treated with SWT before irradiation (clear bars). The error bars represent the standatd deviation (*p<0.05).

In the second regimen analyzed, the animals were treated with SWT or PBS both before irradiation and after irradiation until one day before euthanasia. In the irradiated mice treated with PBS, significant decreases (*p<0.05) were observed in total WBC and granulocyte counts at all of the time points analyzed, in lymphocyte counts at 4, 13, 16 and 18 days post-irradiation and in monocyte counts at 10, 16 and 18 days post-irradiation, as compared to the total WBC, lymphocyte and the monocyte counts in the non-irradiated mice treated with PBS (Table 2). The mice treated with SWT before and after irradiation exhibited statistically significant increases in the total WBC and lymphocyte counts on day 10 post-irradiation, granulocyte counts on day 16 post-irradiation and monocyte counts on day 4 post-irradiation, compared with the corresponding counts in irradiated mice treated with PBS; these counts were not significantly different from the corresponding counts in non-irradiated control mice treated with PBS (Table 2). A direct comparison of lymphocyte and granulocyte counts between irradiated mice treated with PBS and irradiated mice treated with SWT revealed that both lymphocyte- and granulocyte- counts were higher in irradiated mice treated with SWT, but they were statistically significant only for lymphocyte counts at 16 and 18 days post-irradiation (Figure 2).

**Table 2:** Leukocyte counts determined in non-irradiated mice treated with PBS (PBS) and irradiated mice treated with PBS (IRR+PBS) or SWT (IRR+SWT) before and after irradiation

Cells (× 10^3^/μl)	Treatment	4 days	10 days	13 days	16 days	18 days

WBC	PBS	9.90 ± 1.76	8.00 ± 3.61	7.20 ± 0.46	7.30 ± 1.90	8.40 ± 2.59
	IRR+PBS	2.40 ± 0.57*	4.10 ± 0.76*	3.20 ± 1.38*	3.50 ± 0.54*	3.56 ± 1.09*
	IRR+SWT	2.50 ± 0.50*	4.60 ± 1.26	3.50 ± 1.32*	4.50 ± 1.16*	4.26 ± 097*
Lymphocytes	PBS	7.33 ± 1.07	5.80 ± 3.22	5.81 ± 0.37	6.00 ± 1.61	6.20 ± 1.65
	IRR+PBS	1.60 ± 0.30*	2.94 ± 0.58	2.44 ± 1.24*	2.80 ± 0.61*	2.84 ± 0.80*
	IRR+SWT	1.82 ± 0.38*	3.30 ± 0.95	2.85 ± 1.17*	3.70 ± 0.76*	3.60 ± 0.77*
Granulocytes	PBS	2.52 ± 0.76	2.01 ± 0.67	1.30 ± 0.32	1.19 ± 0.28	2.10 ± 1.05
	IRR+PBS	0.61 ± 0.13*	1.04 ± 0.23*	0.63 ± 0.34*	0.67 ± 0.06*	0.68 ± 0.34*
	IRR+SWT	0.43 ± 0.05*	1.20 ± 0.34*	0.63 ± 0.22*	0.75 ± 0.38	0.64 ± 0.22*
Monocytes	PBS	0.47 ± 0.65	0.18 ± 0.08	0.09 ± 0.03	0.12 ± 0.05	0.14 ± 0.08
	IRR+PBS	0.17 ± 0.15	0.09 ± 0.03*	0.05 ± 0.03	0.04 ± 0.01*	0.04 ± 0.01*
	IRR+SWT	0.04 ± 0.01	0.10 ± 0.02*	0.02 ± 0.02*	0.04 ± 0.01*	0.05 ± 0.01*

* *p* values < 0.05 for comparison with samples from non-irradiated mice treated with PBS.

For a comparison of the two regimens of SWT administration, total WBC, lymphocyte and granulocyte counts from the irradiated mice treated with SWT before or before and after irradiation were divided by the respective blood cell counts in the irradiated mice treated with PBS at the matching time points and compared to a no effect value of 1. The results demonstrated that the relative level of WBC (Figure 4), lymphocyte (Figure 4) and granulocyte (Figure 5) counts in the irradiated mice + SWT, although not always statistically significant, were higher in the group treated with SWT before irradiation than in the group treated with SWT before and after irradiation for all time points evaluated, which indicates that the protective effect of SWT was less prominent when administered both before and after irradiation than when SWT was administered before, but not after, the radiation exposure.

**Figure 3:**
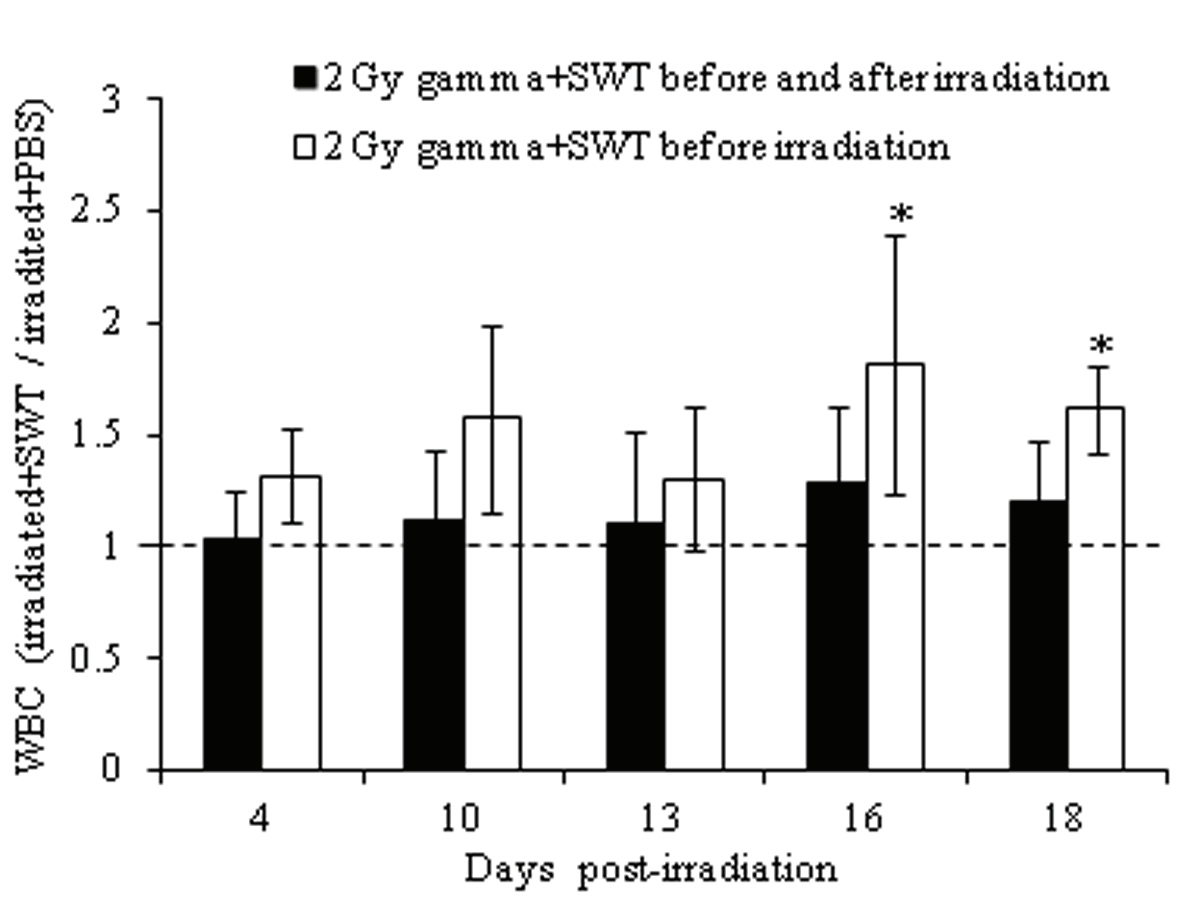
Relative level of WBC counts for irradiated mice treated with SWT before (black bars) or before and after (clear bars) irradiation. *p<0.05 by a t-test in which the results for the irradiated mice treated with SWT before or before and after have been compared to those from the irradiated mice treated with PBS. The error bars represent the standard deviation, whereas the dotted line represents the relative level of WBCs for irradiated mice treated with PBS, which is the no-effect value.

**Figure 4:**
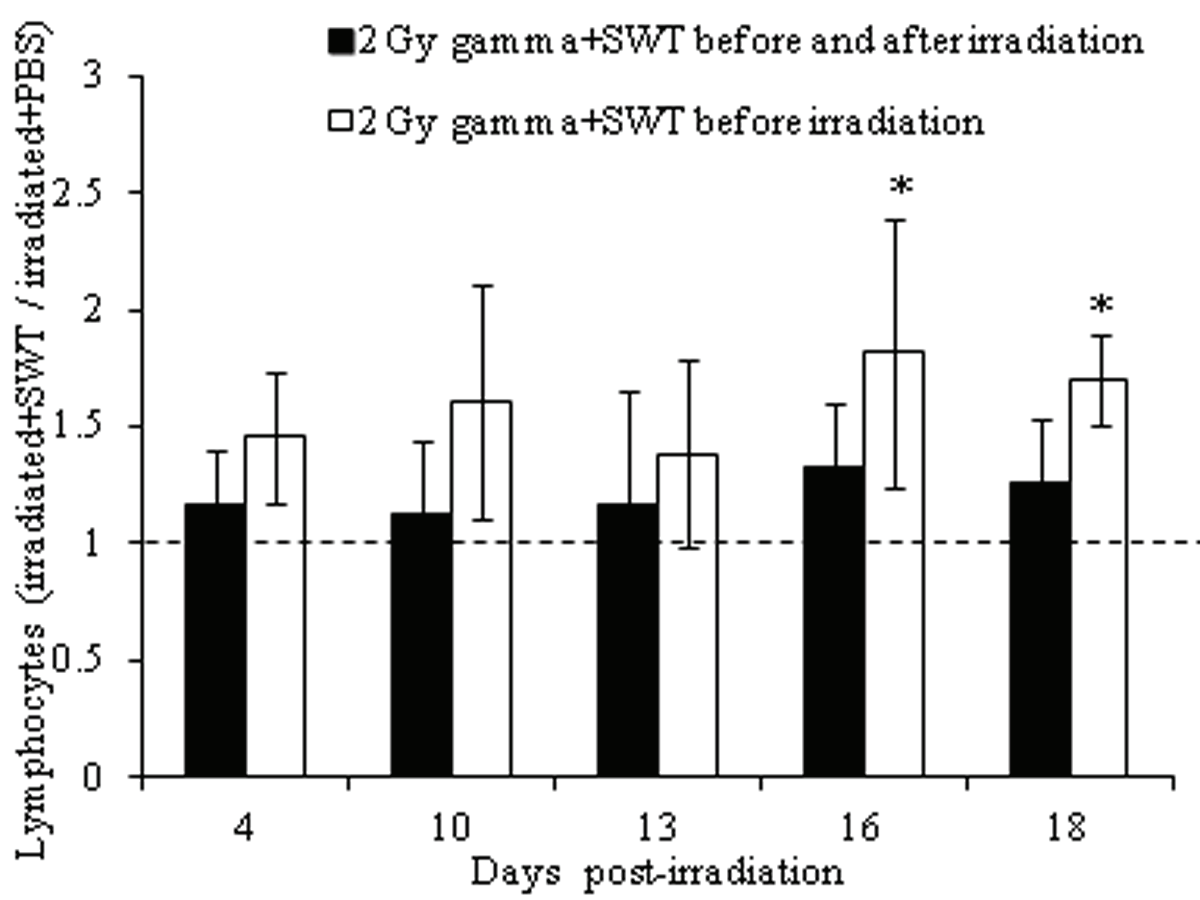
Relative level of lymphocyte counts for irradiated mice treated with SWT before (black bars) or before and after (clear bars) irradiation. *p<0.05 by a t-test in which the results for the irradiated mice treated with SWT before or before and after have been compared to those from the irradiated mice treated with PBS. The error bars represent the standard deviation whereas the dotted line represents the relative level of lymphocytes for irradiated mice treated with PBS, which is the noeffect value.

**Figure 5:**
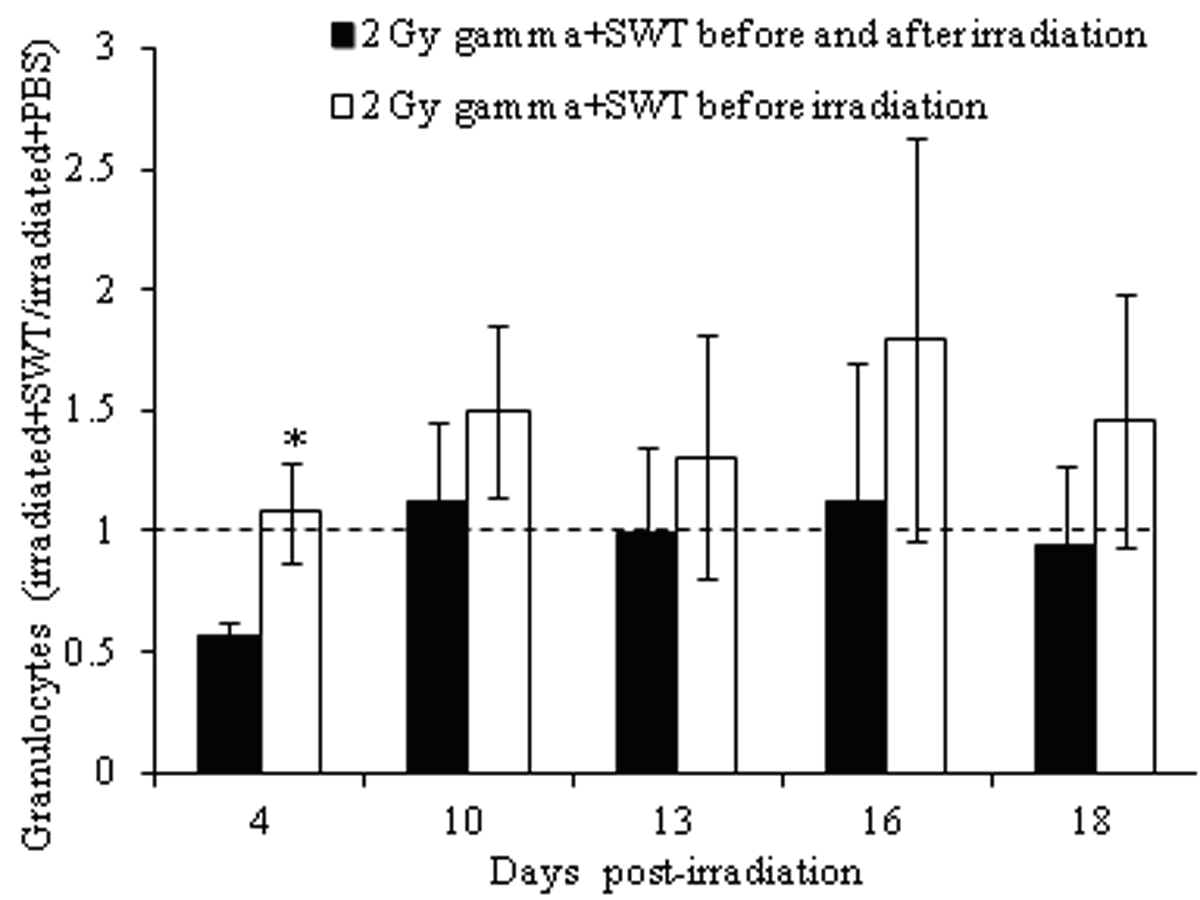
Relative level of granulocyte counts for irradiated mice treated with SWT before (black bars) or before and after (clear bars) irradiation. *p<0.05 by a t-test in which the results for the irradiated mice treated with SWT before or before and after have been compared to those from the irradiated mice treated with PBS. The error bars represent the standard deviation, whereas the dotted line represents the relative level of granulocytes for irradiated mice treated with PBS, which is the no-effect value.

### Effects of SWT on Intestinal Mucosa

**Figure 6:**
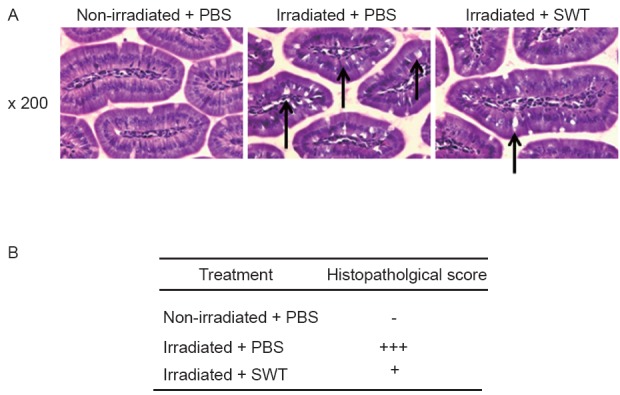
A) Representative images of histological changes in the small intestinal mucosa of mice on day-20 after irradiation; the tissues have been stained with H&E at 200× magnification. The panels in the figure are as follows: Non-irradiated control mice treated with PBS (far left panel), irradiated mice treated with PBS (middle panel) and irradiated mice treated with SWT (far right panel). The arrows point to the changes; B) Histopathological scoring system used for the analyses of H&E sections, as described in the Material and Methods section.

H&E histological staining of sections from non-irradiated or irradiated mice treated with PBS or irradiated mice treated with SWT from day-7 before irradiation until one day before irradiation were prepared with the ileum obtained from the small intestine of mice on day-20 post-irradiation: three sections per mice were examined and scored under a light microscope at 200× magnification (Figure 6). In non-irradiated control mice treated with PBS, the intestinal mucosa was intact and the villi were aligned in an orderly fashion without morphological abnormalities in the epithelial cells (Figure 6A, left column). For the intestines of irradiated mice treated with PBS, the intestinal epithelial cells were loosely lined up and a large number of mucinous cells were observed with a spotted distribution of necrotic cells and cells with a condensed nucleus (Figure 6A, middle column). The morphology of the intestinal mucosa from the irradiated mice treated with SWT before irradiation resembles that of the control animals; only a small number of mucinous cells and few necrotic cells and/or cells with condensed nucleus were observed (Figure 6A, right column). The histopathological scores indicating grades of severity in the morphological changes observed, as described in the Material and Methods section, are shown in Figure 6B. In irradiated mice treated with SWT, the score was two grades lower than the scores for the irradiated mice treated with PBS.

### Effects of SWT on Immunohistochemical Staining of Fibrinogen in Control and Irradiated Mice

**Figure 7:**
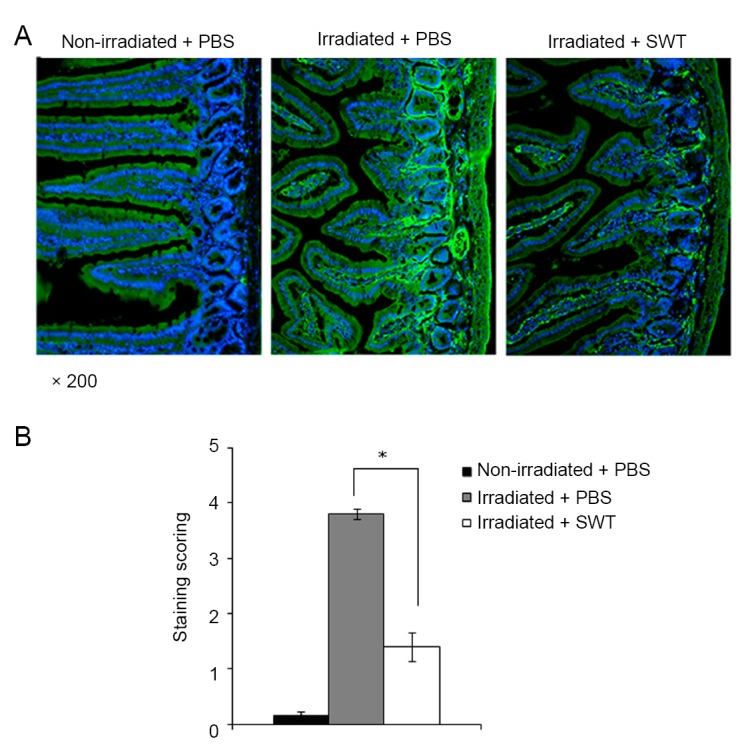
A) Representative immunohistochemical staining images of fibrinogen in tissue sections of small intestine from non-irradiated control mice treated with PBS (far left panel), irradiated mice treated with PBS (middle panel) and irradiated mice treated with SWT (far right panel) on day 20 post-irradiation at 200× magnification, fibrinogen staining is shown as the lightest green color; B) Fibrinogen staining intensity was determined as described in the text (*p<0.005).

The immunohistochemical staining for fibrinogen and pathological analysis was performed on five slides per mouse. Analysis of these slides revealed distinctive differences among the non-irradiated mice treated with PBS, which was included in the experiment as a control group, and irradiated mice treated with PBS or SWT (Figure 7A). The animals in the irradiated group treated with PBS (Figure 7A, middle column) displayed extensive and intense perivascular staining (lightest green) when the results were compared to the animals in the control group (Figure 7A, left column), which did not exhibit fibrinogen staining. For the irradiated group treated with SWT (Figure 7A, right column), perivascular staining of fibrinogen was much less pronounced as compared to the irradiated group treated with PBS. According to the fibrinogen immunostaining intensity determined using the NIH Scion Imaging Scoring software for densitometry analysis, as described elsewhere (14), the differences observed for the values obtained from the irradiated + SWT treatment group compared to those obtained from the irradiated group treated with PBS were statistically significant (Figure 7B).

## DISCUSSION

Although a great amount of effort and resources have been devoted to the discovery and development of radioprotective agents, only a few compounds have reached stages that can be used clinically as effective treatments for radiation. Growth factors have been utilized to increase the numbers of granulocytes and monocytes reduced in animals by radiation exposure (15, 16). Finding compounds that mitigate the toxic effects of radiation in lymphocytes has been less successful, In contrast to synthetic radioprotective agents, natural herbal medicinal products and health foods may provide a feasible alternative treatment. A variety of herbal medicinal products have gained wide acceptance and been used for extremely long periods of time in China for the treatment of various acute and chronic diseases, and some of them have been shown to be capable of alleviating symptoms and discomfort associated with radiation exposure (17). In more recent decades, many formulations made from spray-dried powders of hot water extracts of various mixtures of herbal plants became available as traditional Chinese medicinal products. SWT is one such Chinese medicinal product with a long history of clinical use in traditional Chinese medicine; the uses of SWT have included the alleviation of sickness and discomfort associated with radiation exposure (7, 18). The present study was aimed at evaluating the effects of SWT on the numbers of the different types of WBCs and the intestinal integrity after radiation exposure. The results of the studies reported here on the numbers of different types of WBCs indicate that SWT had radioprotective effects when given before or before and after the radiation exposure, but the SWT treatment given before irradiation had considerably greater radioprotective effects than SWT treatment given both before and after the radiation exposure. The results demonstrated that oral administration of SWT before irradiation was able to delay the decline, and speed up the recovery of lymphocytes, as measured by peripheral lymphocyte counts. These results are noteworthy considering that there is essentially no countermeasure for the reduced numbers of circulating lymphocytes following the exposure of animals to significant doses of radiation.

As a common treatment modality for cancer in the clinic, radiation therapy plays a crucial role in the curative treatment of cancers and the palliation of symptoms associated with advanced malignant diseases. Gastrointestinal side effects, such as vomiting and diarrhea, limit the utility of this important therapeutic modality; these side effects can significantly reduce the quality of life for cancer patients and add an extra burden to the cost of health care. In some cases, the radiation side effects have resulted in patients refusing to continue their radiotherapy, sometimes with fatal consequences. Acute radiation intestinal injury occurs primarily as a result of mitotic and apoptotic cell death in the crypt epithelium, resulting in insufficient replacement of the surface epithelium of the intestinal villi. Doses as low as 1 Gy can cause breakdown of the epithelial barrier, which may result in the penetration of antigens, bacterial products, and digestive enzymes from the intestinal lumen into the intestinal wall (19). Zhou *et al* (20) have reported that total body gamma irradiation of mice at a 2 Gy dose causes breaks in the epithelial lining of mouse intestines and allows the translocation of bacterial products into the circulation; the translocation of bacterial products such as lipopolysaccharide (LPS) to organs outside the GI tract could greatly contribute to the radiation sickness symptoms which are observed in people exposed to low doses of ionizing radiation. At moderate radiation doses, there is hypoplasia of the mucosa, while higher doses cause frank ulcerations or areas of complete epithelial denudation, which results in inflammation of the intestinal mucosa (mucositis). Therefore, it is imperative to find mitigators for the intestinal injury caused by radiation exposure. The results from analyses of the H&E stained intestinal tissue presented here suggest that irradiation of mice with a low dose of gamma rays caused the presence of mucinous and necrotic cells, as well as cells with a condensed nucleus, which suggest the breakdown of the cells in the intestinal barrier, while the results of fibrinogen immunohistochemical staining indicated that mice irradiated with a 2 Gy dose of gamma rays displayed massive perivascular fibrinogen immunostaining, which suggests an inflammatory process since fibrinogen is an established marker of inflammation (21). Treatment of irradiated mice with SWT had radioprotective effects in the intestinal epithelial cells and the intestinal inflammatory processes resulting from the exposure of mice to the 2 Gy dose of radiation. In summary, the results presented here indicate that SWT is a potential mitigating agent for the reduction in the numbers of circulating lymphocytes and the adverse effects on the intestinal lining in irradiated mice. The mechanism(s) by which SWT exerts these effects, as well as the determination of the SWT component(s) responsible for these effects, remain to be determined.
